# The Relationships of Human-Cobot Interaction Fluency with Job Performance and Job Satisfaction among Cobot Operators—The Moderating Role of Workload

**DOI:** 10.3390/ijerph20065111

**Published:** 2023-03-14

**Authors:** Mateusz Paliga

**Affiliations:** Institute of Psychology, Faculty of Social Sciences, University of Silesia in Katowice, 40-007 Katowice, Poland; mateusz.paliga@us.edu.pl

**Keywords:** cobot, collaborative robot, human-robot interaction, fluency, job performance, task performance, organizational citizenship behavior, creative performance, job satisfaction, workload

## Abstract

Modern factories are subject to rapid technological changes, including the advancement of robotics. A key manufacturing solution in the fourth industrial revolution is the introduction of collaborative robots (cobots), which cooperate directly with human operators while executing shared tasks. Although collaborative robotics has tangible benefits, cobots pose several challenges to human-robot interaction. Proximity, unpredictable robot behavior, and switching the operator’s role from a co-operant to a supervisor can negatively affect the operator’s cognitive, emotional, and behavioral responses, resulting in their lower well-being and decreased job performance. Therefore, proper actions are necessary to improve the interaction between the robot and its human counterpart. Specifically, exploring the concept of human-robot interaction (HRI) fluency shows promising perspectives. However, research on conditions affecting the relationships between HRI fluency and its outcomes is still in its infancy. Therefore, the aim of this cross-sectional survey study was twofold. First, the relationships of HRI fluency with job performance (i.e., task performance, organizational citizenship behavior, and creative performance) and job satisfaction were investigated. Second, the moderating role of the quantitative workload in these associations was verified. The analyses carried out on data from 200 male and female cobot operators working on the shop floor showed positive relationships between HRI fluency, job performance, and job satisfaction. Moreover, the study confirmed the moderating role of the quantitative workload in these relations. The results showed that the higher the workload, the lower the relationships between HRI fluency and its outcomes. The study findings are discussed within the theoretical framework of the Job Demands-Control-Support model.

## 1. Introduction

Contemporary manufacturing industry is subject to an evolution in which the interaction between humans and technology is fundamental. The emergence of advanced human-robot interaction (HRI) has modified the performance of tasks [1] to boost system productivity and efficiency [2]. Importantly, because workers play a strategic role in manufacturing activities [3], their abilities and skills are enhanced rather than replaced by robotic technology [4,5] in human-centric automation [6]. In order to empower operators, enabling technologies are implemented in the industry, among which are collaborative robots [7].

Collaborative robots (cobots) are designed for mutual and direct interaction with humans [8]. Because they are equipped with safety assurance mechanisms [9], cobots assist operators by delivering and handling objects [10] in a fenceless and confinement-free environment. Direct human-robot interaction aims at incorporating the unique advantages of humans [11] and collaborative robots [12] by combining the skills and abilities of both parties to achieve a common goal [13] in a temporospatial overlap [14]. Cobots take on strenuous and repetitive tasks [15,16] by providing stamina and strength [17] while human operators are responsible for handling unexpected events [11] due to their resources of flexibility and cognitive and soft skills [7]. Hence, human-cobot interaction results in a synergic combination of the capabilities of humans and their robotic counterparts [18].

Integrating cobots in manufacturing processes is viewed as significantly beneficial. Due to the increased job attractiveness through the means of human-robot interaction, organizations deploying collaborative robots can offset the shortage in the industry workforce and guarantee jobs at a certain site without the necessity to move the production elsewhere [19]. For operators, automated robotic systems can help prevent adverse health effects [16] during work in unsafe environments [20]. Generally, the surge of interest in collaborative robots stems from the fact that they are seen as technological solutions for improving performance [1,7,21]. More than that, however, scholars and practitioners suggest that cobots enhance operators’ well-being [1,22].

In the ongoing studies on the positive consequences of cobot implementation, researchers advocate for the need to consider human factors, as they are requisite conditions for human-robot interaction to be fruitful. Focusing on factors such as perceived safety, trust, anxiety, mental strain, stress [11,15,23], or robot acceptance [12] can help understand what makes operators feel comfortable and efficient [24,25]. Contrarily, the omission of these elements can result in operators’ frustration [7,24,26] and failure of human-robot system performance [14]. Hence, the first aim of this research is to verify the relationships of cognitive, emotional, and behavioral aspects of fluency in human-robot interaction with job performance (i.e., task performance, organizational citizenship behavior, and creative performance) and job satisfaction. Based on the literature search and to the author’s best knowledge, such an aim has not yet been realized in the empirical studies among cobot operators. The assumption behind this aim is that successful HRI is affected by humans’ cognitive and emotional processes [13] as well as behavior [27]. Therefore, subjectively perceived fluency in human-robot interaction can be an important factor for operators’ performance and attitudes during collaboration with a cobot.

The second aim of this study is the exploration of quantitative workload as a moderator in the relationships of HRI fluency with job performance and job satisfaction. The impetus for such exploration is the general notion that the direction and magnitude of the effects of robot deployment on operators are theoretically ambiguous and need further empirical verification [16]. Specifically, although the research on the positive impact of cobots on workers’ experiences has been prolific, the assumption that collaborative technology is exclusively advantageous would be biased. Although many studies suggest that cobots support humans in terms of physical and mental load [1,9,10,18], there are other empirical investigations showing that cobots increase workload [7,9,10,15,28]. The conflicting pattern of findings indicates that there is still a need to explore workload [22,24] as an essential individual factor in human-robot interaction [9]. In fact, the evaluation of workload in human-machine interaction is a crucial point when investigating satisfaction and performance [29], which is the general aim of the presented research.

The remainder of the paper unfolds as follows. Section 2 presents the theoretical background regarding human-robot interaction fluency, job performance, job satisfaction, and workload. Section 3 describes the current study aims, hypotheses, and their rationale. Next, the pool of participants and used measures are characterized in Section 4, followed by the description of statistical analyses in Section 5. Finally, in Section 6, the discussion of obtained results is presented with the identification of the strengths and limitations of the study. The paper ends with a concise conclusion in Section 7.

## 2. Theoretical Background

### 2.1. Human–Robot Interaction Fluency

Fluency in human-robot interaction describes a highly coordinated meshing of shared activities between the human and the robot [30]. To achieve fluency, the human and robotic agents must create a well-synchronized and synergic team and perform joint activities [31] at high levels of coordination and adaptation [32,33]. Continuous fluent interaction requires the team members to jointly understand the capabilities of the others [11] and is necessary to efficiently perform shared tasks [15,17] in an intelligent system [34].

Collaborative robots change the working system’s dynamics and increase efficiency [2]. However, bringing a robotic agent into the loop requires a new comprehension of human-robot team effectiveness and fluency as dependent on three sources [35]. The notion of three separate fluency agents, i.e., the operator, the robotic counterpart [32,36,37], and the human-robot team [38] with its fluidity [39] was used by [31], who described a three-dimensional approach to human-robot interaction fluency. The idea was further developed by [40], who described four HRI fluency perspectives: human contribution-oriented, human emotions-oriented, robot-oriented, and team-oriented fluency.

Regarding *human contribution-oriented fluency*, [4,10] point out that the variability and reliability of human behavior affect human–robot interaction in terms of safety, quality, and productivity. Similarly, [27] claim that the operator supports the technical system and production requirements, and [9] even argue that the human component influences the performance of the human-robot team more than the technological one. In essence, to maintain efficient and fluent HRI, human behavior should be considered [41] and investigated. Therefore, human contribution-oriented fluency reflects the operator’s performance of common tasks with the robot, engagement, and input to the human–robot interaction [40].

The *human emotions-oriented fluency* is based on the assumption that people interact with robots as they do with co-workers [10] or people [42] rather than tools due to their willingness to apply social characteristics to technology [43]. Precisely, this perspective reflects the cognitive-affective states evoked in humans during collaborating with a robot. Hence, the core of this fluency is the operator’s feelings about the robot’s commitment to the teamwork and his or her cognitive or affective trust toward the robot [31,40]. Trust is a major prerequisite and a crucial challenge to overcome for the success of human–robot teamwork [4,44]. As a human factor [35], it depends on the reliability [11] and predictability of the robotic agent’s performance [17], and the fulfillment of human expectations [45]. When high, trust leads to the operators increasing their reliance on robots and reducing supervision over them [46], but when low, it may encourage robot underutilization and drops in performance [13].

If the robot is to become a factual and co-responsible member of the human-robot team, its performance of joint tasks has to be visible and directable by the human [34]. Based on this, [31] introduced the idea of *robot-oriented fluency*, which refers to the operator’s perception and evaluation of the robot. While collaborating with the human, the robot realizes its part of the task and completes or corrects the operator [31], mainly through delivering and handling objects [10]. In general, the robotic member supports the effectiveness of the automated system [27] through device primitives and skills [47] to a similar extent that the operator does. This support, evaluated by the operator, is the core of robot-oriented fluency [40].

As noted by [34], the coordination across agents in the human-robot system is as important as their actions considered separately. Therefore, to directly capture *team-oriented fluency* [30], Paliga and Pollak [31] considered the team level of an automated system committed to fulfilling joint intentions [48] and performing together in a dyad [22] or team [17]. Specifically, effective team structure [11] and coordination [49] are the factors that facilitate fluent human-robot interactions [40]. Hence, team-oriented fluency refers to synchronous or sequential task performance [1] in a temporospatial overlap [14] by two (or more) mutually coordinated team members whose qualities should be utilized according to their advantages [50].

### 2.2. Job Satisfaction

Job satisfaction is the subjective well-being that workers experience at work [51,52]. Specifically, it is a work attitude that comprises multidimensional psychological responses to one’s job [53] with a cognitive component (knowledge, evaluative judgments, and opinions about the job), an affective component (emotions and feelings about the job), and a behavioral component (individual predispositions and manifestations of employee’s actions related to performed work) [54,55]. The subjective assessment of job satisfaction is typically arrayed along a negative-to-positive continuum [56].

For robot operators, as for other incumbents, the feeling of job satisfaction dissuades them from changing their job [52,57], making it relevant not only for the individuals but also for the stability of the automation-based manufacturing system. When considering the general perspective of job automatability, [58] found that its high level was related to lower job satisfaction; however, this was only when workers assessed their work as uninteresting and meaningless. This suggests that it is not automation of work itself but rather monotonicity and lack of task variety that relate to dissatisfaction. On the other hand, [52] argued that lower job satisfaction might stem from incumbents’ fear of being replaced by machines, which echoes the suggestion of [59] that autonomous technology threatens employees’ job security.

Among studies conducted with the use of a collaborative robot, the research by [23] showed that an autonomously operating cobot induced more adverse psychological and physiological reactions than a manually operated cobot. Such a result resonates with the notion that autonomous robots represent a realistic and symbolic threat to the operators’ well-being [60] and can evoke negative emotional and cognitive reactions by taking away the decision-making prerogative from humans [61]. Therefore, to be satisfied, operators should be able to retain the feeling of self-determination and control in human-robot interaction [19]. Finally, an optimistic premise for cobot deployment is the study by [62], who carried out an experiment on human-cobot collaboration and found that both adult and senior collaborators were highly satisfied with the interaction and accepting of the collaborative robot.

### 2.3. Job Performance

Many definitions of job performance have been proposed [63]. A widely accepted one was introduced by [64,65], who defined performance in terms of behaviors and actions presented by employees. In other words, performance refers to what employees do in their workstations [66]. However, this behavioral perspective requires several assumptions. First, because performance is a latent construct that cannot be measured directly [67], it is defined by judgmental and evaluative processes [66] referring to employees’ observable [68] and scalable actions in the workplace [63]. Second, not every action manifests as performance, but only behaviors relevant to organizational goals [64]. Finally, behavior that falls within the performance concept differs from its outcomes [65], such as productivity [67] or effectiveness [68] over which the employees might not have control [66]. Taking everything together, job performance can be defined as employees’ observable and scalable behavior, over which they have control and which is linked with and contributes to organizational goals.

Job performance is a multidimensional construct [64,68], with each dimension having indicators that can be measured directly [67]. The present research investigates three dimensions: task performance, organizational citizenship behavior, and creative performance. Task performance refers to workers’ proficiency in performing activities recognized formally as part of their jobs which contribute to the organization’s technical core [69]. Hence, activities subsumed under the task performance concept are described in the formal job description [70,71], and employees commit to fulfilling them when signing the contract [54].

Organizational citizenship behavior (OCB) is individual behavior that is discretionary, not directly or explicitly recognized by the formal reward system, and that promotes the effective functioning of groups [72] and the organization as a whole [63,73]. To achieve this, employees showing OCB help other organization members perform their jobs or show support for and conscientiousness toward the organization [74]. Examples of behaviors that exceed the formal job description include taking on extra tasks, showing initiative, or sharing knowledge and experience with other employees. Although voluntary, OCB is relevant for task performance (mandatory in its nature) because it shapes the organizational, social, and psychological context in which task activities are performed [69].

Creative performance refers to behavioral manifestations of creativity [67], that is, the creation of products, ideas, procedures, or processes produced by an individual in the organization [75,76]. Significantly, for performance to be creative, it needs to meet the standards of quality, utility, and novelty [75,77]. In the organizational context, employees perceive creativity requirement as the expectancy or need to generate work-related ideas. This perception refers to both explicit requirements and other cues [78].

In the research on human-robot interaction, task performance has been investigated with objective and subjective metrics. Examples of objective measures are: counting the number of parts assembled correctly by the operator [57], measuring task execution times [79], number of units produced per period, the resources consumption for a given output, or the number of defective items produced [7]. Subjective indicators include different scales used for measuring perceived assessment of task accomplishment in experimental studies (e.g., [62]), cross-sectional studies among students (e.g., [31]), or among cobot operators working on the shop floor (e.g., [40]). Regarding organizational citizenship behavior and creative performance, there is a dearth of evidence to rely upon, as these concepts of performance have been neglected in the studies on human-robot interaction. Hence, it has left a void and a need for empirical verification amid cobot operators, which the current study aims to fill.

### 2.4. Workload

Workload represents the volume of work required of an employee [80]. Specifically, quantitative workload pertains to the pace and amount of work an employee performs in a given time period [81,82]. As such, it differs from qualitative workload, which refers to incumbents’ skills and effort necessary to complete the task [83].

The quantitative workload is an objective work demand [82] that requires physical (e.g., motor) and psychological (e.g., cognitive) effort [83,84] from the employee to perform a high amount of work or fast-paced tasks. In the short term, a quantitative workload can have both negative and positive consequences. When faced with a low workload, under-stimulated incumbents can experience boredom and loss of concentration [83]. Contrarily, an overly high workload can leave the employees tense [83] and fatigued [82]. Moreover, [80] note that high workload levels can be followed by workers’ feelings of uncertainty and frustration due to the disruption of goal achievement. Therefore, [83] suggests that an inverted U-shaped relationship of workload with job performance and job attitudes exists. Accordingly, a moderate workload can have a positive effect on employees’ behavior and attitudes due to the optimal activation generated in a motivational process. In the long term, however, the consequences of workload are mostly harmful. Chronic overstimulation by job demands leads to stress [81,85], extreme tiredness and work-related fatigue [82], lower psychological well-being [84], and impaired performance and health [83].

Baumgartner, Kopp and Kinkel [19] point out that cobot implementation may lead to multifaceted changes in workload. In line with their notion, some studies suggested that cobots could relieve humans in terms of physical [9,10,18] and mental load [1], while other research examples showed that deploying cobots led to increased workload [7,9,10,15,28].

## 3. The Study Aims and Hypotheses

The aim of this study is twofold. The first goal is to verify the relationships of four perspectives of fluency in human-cobot interaction with three facets of employees’ job performance and job satisfaction. The assumption behind these relations is that high-quality and fluent HRI increases employee performance [1,24,30], productivity and efficiency of the human-machine system [10,15], and operators’ well-being [22]. Hence, fluency is considered a pertinent factor when investigating HRI [11]. Still, the empirical base of performance-related consequences and correlates of fluency in HRI remains thin, with few study examples having shown that fluency can lead to increased individual task performance [31,40,79] and team performance [30]. Regarding cobot operators’ well-being and satisfaction, the problem remains an open question due to the lack of evidence. However, some theoretical premises can be drawn from the Job Demands-Control-Support (JDCS) model of occupational stress [86,87]. In light of this model, [40] argued that working with a collaborative robot is an active job where high job demands meet high control stemming from psychological safety, predictability, and fluency of interaction with a cognitively and emotionally trustworthy robotic agent. According to [86], the highest job satisfaction occurs specifically with active jobs. Furthermore, cobots are thought to be co-workers rather than tools [10,40] and can therefore offer social support to their human operators, which is an important factor for work-related well-being and job satisfaction [86,88]. Based on the theoretical premise and empirical investigations, the following hypotheses were formulated:

**Hypothesis 1.**  *Fluency in human-cobot interaction (i.e., human contribution-oriented, human emotions-oriented, robot-oriented, and team-oriented fluency) will be positively related to employees’ job performance (i.e., task performance, organizational citizenship behavior, and creative performance).*

**Hypothesis 2.**  *Fluency in human-cobot interaction (i.e., human contribution-oriented, human emotions-oriented, robot-oriented, and team-oriented fluency) will be positively related to employees’ job satisfaction.*

The second aim of this study is to explore the moderating role of quantitative workload while interacting with a cobot in the relationships of HRI fluency with job performance and job satisfaction. The research on workload among robot operators has been ambiguous or even conflicting, with studies suggesting that robot deployment either decreases or increases workload. Generally, the increase in a cobot operator’s workload can be on-the-job physical or mental. The first phenomenon stems from the reduction of the work time cycle and the consequent requirement for operators to move faster [28]. The latter can be an effect of anxiety or lack of expertise and confidence in interacting with robots [15], the execution of time-sharing activities [21], switching the operator’s role from a co-operant to a supervisor [21,89], repeatability of the operator’s movements [10], or the lack of inclusion of participants’ preferences for the task shared with a robot [31,90].

The relationships of workload with job performance and job attitudes (e.g., job satisfaction) are suggested to be an inverted U-shape [83], which means that both low and high workload levels can have adverse effects on employees. Specifically, while under-stimulation can cause boredom and loss of concentration [83], a high workload may lead to tension, fatigue and tiredness, frustration, stress, and lower well-being and performance [80,81,82,83,84,85]. This holds true also for cobot operators [21]. Contrarily, a moderate workload can motivate operators by providing adequate stimulation for their resources and, thus, optimal activation [83]. The complex pattern of relations between workload and other work experiences begs the question of whether workload can moderate the relationship of fluency in HRI with job performance and job satisfaction. Therefore, the following hypotheses were formulated:

**Hypothesis 3.**  *Quantitative workload will moderate the relationship between overall fluency in human-cobot interaction and job performance (i.e., task performance, organizational citizenship behavior, and creative performance).*

**Hypothesis 4.**  *Quantitative workload will moderate the relationship between overall fluency in human-cobot interaction and job satisfaction.*

It is essential to verify the hypotheses and investigate how the integration of collaborative technology changes human workers’ experience [13,91]. In particular, there is a need to study human factors [11] and the psychophysical state of operators [9] since elements like emotions and cognitions can influence HRI and performance [13]. Therefore, the cognitive, emotional, and behavioral aspects of subjective HRI fluency should be analyzed in relation to such key topics as job performance [40], satisfaction [7,91,92], and workload [9,22,24,29], which require further investigation in light of the research on human factors.

## 4. Materials and Methods

### 4.1. Participants and Procedure

This online survey study was conducted among operators working daily with collaborative robots on the shop floor. All participants were informed about the aim of the study. Participation in the study was voluntary. Informed consent was obtained from all participants, and the data collected were anonymized and kept confidential. The institutional ethics committee of the University of Silesia in Katowice approved this research project (decision number KEUS 125/05.2021). The study was carried out according to the principles of the Declaration of Helsinki and the recommendations of the National Science Centre in Poland regarding studies involving human participation.

Two hundred professional cobot operators took part in the study (120 men and 80 women). The age of the respondents ranged from 23 to 55 years, averaging at 37.93 years (±6.26). Regarding participants’ education level, most of them (91%) held a higher education degree, 8.5% completed upper secondary education, and only 0.5% achieved a primary education level. The operators had diverse seniority in robot collaboration, which varied between 3 months and 35 years, with the average duration at 6.32 years (±5.46). Several different types of cobots were included in the study. Examples comprise robots produced by KUKA (HRC-compatible LBR intelligent industrial work assistant (iiwa) 7 R800, R800 CR, and 15 R820, Yamaguchi, Japan), Universal Robots (collaborative robot arms such as UR3/UR3e, UR5/UR5e, UR10/UR10e, Odense, Denmark), FANUC (the “Green robot” family of examples of CR-4iA, CR-7iA, CR-7iA/L, CR-14iA/L, CR-15iA, and CR-35iA as well as the CRX Collaborative Robot Series examples of CRX-10iA and CRX-10iA/L, Yamanashi, Japan), ABB (collaborative industrial robot YuMi, Zürich, Switzerland), Yaskawa (the human-collaborative robot arm MOTOMAN HC20DT, Fukuoka, Japan), Mitsubishi Electric Collaborative Robot (Melfa Assista, Tokyo, Japan), Kawasaki (dual-arm collaborative robot duAro, Tokyo, Japan), and collaborative robotic arms produced by Epson and OMRON.

### 4.2. Measures

The Fluency in Human–Robot Interaction The scale developed by [40] was used to measure four perspectives of fluent interaction between the operators and their robotic counterparts. The tool comprises 12 questions regarding human contribution (e.g., “I contributed to the fluency of the interaction”), human emotions (e.g., “I trusted the robot to do the right thing at the right time”), robot (e.g., “The robot did its part successfully”), and team-oriented fluency (e.g., “The human–robot team worked fluently together”). The respondents answered the questions on a seven-point Likert scale (1 = *I strongly disagree* to 7 = *I strongly agree*). The obtained data were used to confirm a second-order factor model with four perspectives of fluency in HRI as subscales and a general (overall) score. Results of confirmatory factor analysis (χ^2^ = 82.47; df = 50; *p* < 0.01; RMSEA = 0.06; 95% CI = 0.03–0.08; CFI = 0.98; NFI = 0.96; SRMR = 0.03) proved that the data fitted the model well [93]. Cronbach’s alphas ranged from 0.85 to 0.91 for the subscales, while the reliability of the whole scale was 0.96.

Task performance was measured using three items from [70] (e.g., “I fulfill all the requirements of the job’’) with a seven-point Likert response scale (1 = *I strongly disagree* to 7 = *I strongly agree*). The participants were instructed to specifically indicate their answers regarding performance during work with the cobot. The score was computed by averaging the responses in all three items. Cronbach’s *α* was 0.87.

Organizational citizenship behavior was assessed with three items from [94] (e.g., “I willingly share expertise, knowledge, and information to help improve the effectiveness of others in my work group”). The operators indicated their behavior using a seven-point Likert scale (1 = *I strongly disagree* to 7 = *I strongly agree*). An average score was used with Cronbach’s *α* = 0.86.

Creative performance was measured with the three items created by [75] (e.g., “At work with a robot, I am creative: I develop original ideas for my organization”). The respondents answered the questions on a seven-point Likert scale (1 = *Not at all* to 7 = *To a great extent*). An average score was calculated, and Cronbach’s *α* was 0.91.

The job satisfaction scale by [95] was used to measure operators’ judgments and opinions about their work with a cobot. The scale comprises five items (e.g., “I am satisfied with my work with the robot”) with a seven-point Likert response scale (1 = *I strongly disagree* to 7 = *I strongly agree*). The score was computed by averaging the responses in all five items. Cronbach’s *α* was 0.91.

The quantitative workload was indicated by the respondents using the Quantitative Workload Inventory [80]. The tool consists of 5 items (e.g., “How often does your job require you to work very fast”) with a five-point Likert response scale (1 = *Less than once per month or never* to 7 = *Several times per day*). An average score was computed from the answers to all five items. Cronbach’s *α* = 0.93.

## 5. Results

The statistical analyses (calculation of descriptive statistics, correlations, and regression models) were carried out in IBM^®^ SPSS^®^ Statistics (version 28.0.1.0) and moderation analysis was conducted in PROCESS macro (version 3.5) for IBM^®^ SPSS^®^ Statistics on the data from 200 respondents. 

### 5.1. Descriptive Statistics

Descriptive statistics (shown in Table 1) indicate that the operators’ subjectively perceived fluency in human-robot interaction was moderately high to high. The same can be seen for different aspects of job performance: task performance, organizational citizenship behavior, and creative performance were rated as moderately high to high. A similar situation was found in the case of job satisfaction. Finally, the quantitative workload at work with a cobot was averagely assessed by the operators as moderately low to moderate.

The Kolmogorov–Smirnov normality test results were significant in all cases (Table 1). However, the values of skewness and kurtosis were low and did not exceed 0.89 and 0.47, respectively. Existing literature [96,97,98] suggests that in such cases, the obtained skewness and kurtosis prove normal univariate distribution. Hence, the assumptions of further analysis were not violated.

### 5.2. Correlation and Regression Analyses

Correlation and regression analyses were carried out to verify Hypotheses 1 and 2 concerning the relationships of fluency in human-robot interaction with job performance and job satisfaction. Table 2 shows the results of the correlation analysis.

The patterns of correlations are consistent with the theoretical assumptions and predictions made in this study. All perspectives of fluency in human-robot interaction, as well as overall fluency, correlate positively and strongly with all facets of job performance (i.e., task performance, organizational citizenship behavior, and creative performance) and job satisfaction. Furthermore, different types of performance are in positive and strong relations with job satisfaction.

Since all perspectives of fluency in HRI are correlated (see Table 2), hierarchical regression analysis was performed to further verify their relationships with job performance and job satisfaction. It was carried out to investigate the unique contribution of each perspective of fluency as a predictor [99,100]. The results are presented in Table 3.

Regarding task performance, it is predicted by human emotions-oriented, robot-oriented, and team-oriented fluency. All these perspectives of fluency are in positive relationships with task performance, and together they explain 60% of its variance. For organizational citizenship behavior and creative performance, the only significant and positive predictor is team-oriented performance, which allows for predicting 64% and 54% of the variance of the dependent variables, respectively. Lastly, job satisfaction can be predicted with human contribution-oriented, human emotions-oriented, and team-oriented performance. Together, these positive predictors account for 80% of the variance in job satisfaction.

### 5.3. Moderation Analysis

To test Hypotheses 3 and 4 about whether the qualitative workload moderates the relationships of fluency in human-robot interaction with three facets of job performance and job satisfaction, a series of moderation analyses were conducted. In each analysis, overall fluency in HRI was the predictor, the qualitative workload was the moderator, and either a facet of job performance or job satisfaction was the dependent variable. The analysis was carried out on standardized data using a bootstrap method with 10,000 replications, indicating 95% confidence intervals. The results of the moderation analysis are shown in Table 4.

**Figure 1:**
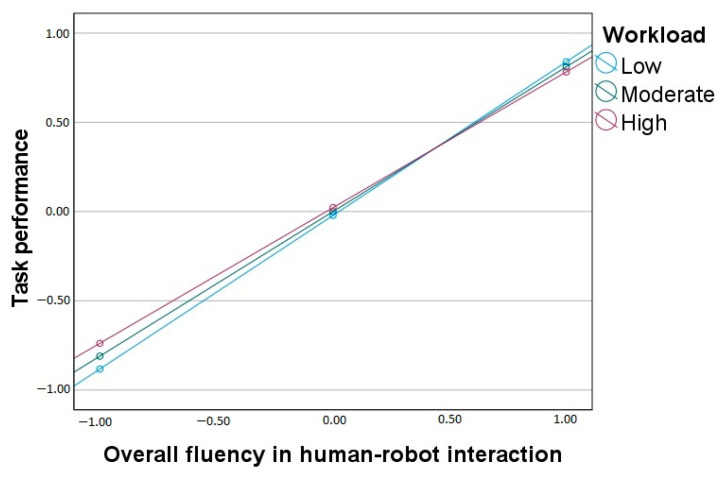
Workload as a moderator between overall fluency in human-robot interaction and task performance.

For task performance, the overall model is statistically significant (*F*(3, 196) = 141.56, *p* < 0.001, R^2^ = 0.68). Overall fluency in HRI is positively related to task performance, but workload does not moderate its effect on task performance (the change in explained variance is insignificant). The insignificant interaction effect is depicted in Figure 1. The standardized slopes for the effect of overall fluency on task performance are significant and similar at low (*β* = 0.86), moderate (*β* = 0.81), and high (*β* = 0.76) levels of workload.

**Figure 2:**
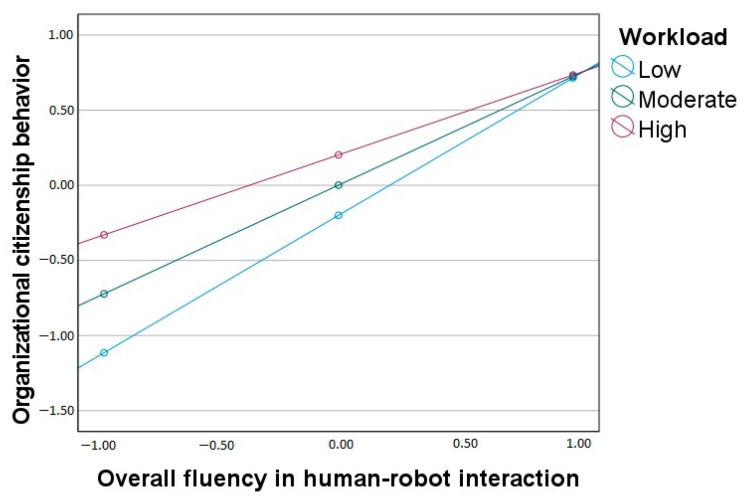
Workload as a moderator between overall fluency in human-robot interaction and organizational citizenship behavior.

Regarding organizational citizenship behavior (OCB), the overall model is statistically significant (*F*(3, 196) = 123.52, *p* < 0.001, R^2^ = 0.65). Overall fluency in HRI is in a positive relation with OCB, and workload significantly moderates the effect of overall fluency. The interaction effect, presented in Figure 2, shows that the standardized slopes for the effect of overall fluency on OCB are significant at low (*β* = 0.91), moderate (*β* = 0.72), and high (*β* = 0.53) levels of workload, but the gradient becomes lower with the increased workload.

**Figure 3:**
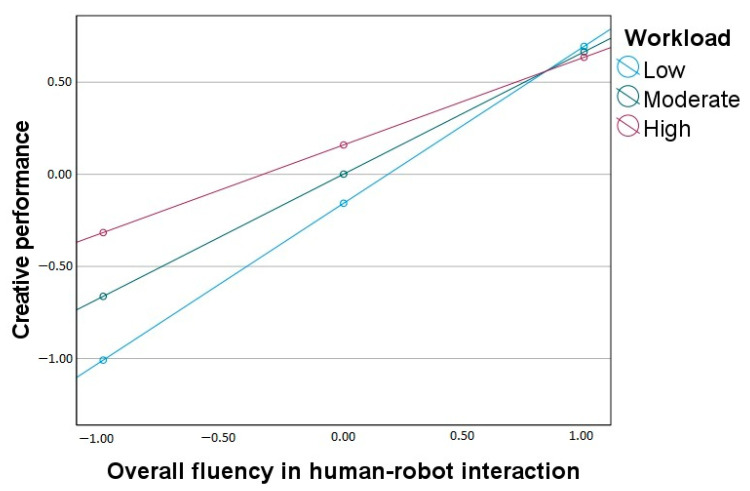
Workload as a moderator between overall fluency in human-robot interaction and creative performance.

Concerning creative performance, the overall model is also statistically significant (*F*(3, 196) = 80.76, *p* < 0.001, R^2^ = 0.55). Overall fluency in HRI positively correlates with creative performance, and workload significantly moderates the effect. The interaction effect is shown in Figure 3. The standardized slopes for the effect of overall fluency on creative performance are significant at low (*β* = 0.85), moderate (*β* = 0.66), and high (*β* = 0.48) levels of workload, yet the gradient becomes lower with the increased workload.

**Figure 4:**
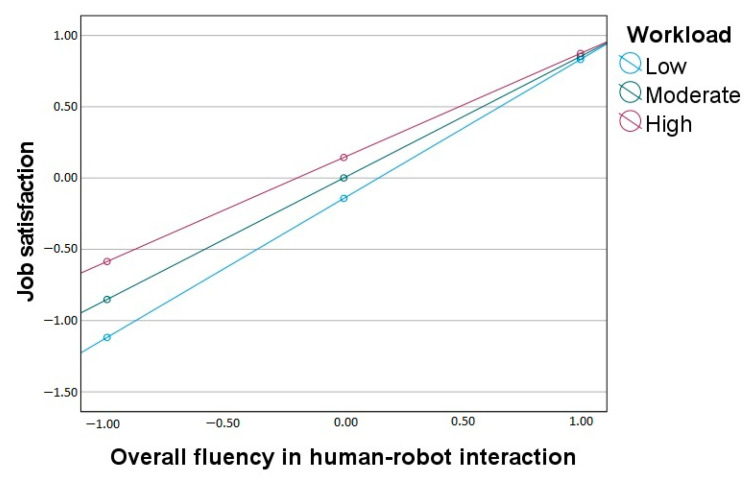
Workload as a moderator between overall fluency in human-robot interaction and job satisfaction.

Finally, for job satisfaction, the overall model is statistically significant (*F*(3, 196) = 283.89, *p* < 0.001, R^2^ = 0.81). Overall fluency in HRI is positively related to job satisfaction, and workload moderates the effect significantly. As depicted in Figure 4, the standardized slopes for the effect of overall fluency on satisfaction are significant at low (*β* = 0.98), moderate (*β* = 0.85), and high (*β* = 0.73) levels of workload, but again the gradient becomes lower with the increased workload.

To sum it up, as workload increases, the strength of the relationships of overall fluency in HRI with organizational citizenship behavior, creative performance, and job satisfaction decreases. However, the moderating effect is not observed for task performance as a dependent variable.

## 6. Discussion

This empirical study sought to investigate the relationships of fluency in human-robot interaction with three facets of job performance and job satisfaction. The second aim was to verify the moderating role of quantitative workload in these relationships. The presented research was a survey study conducted on a pool of 200 cobot operators who interact with collaborative robots on a daily basis.

The obtained results show that fluent human-robot interactions are positively related to employees’ task performance, organizational citizenship behavior, and creative performance. Specifically, the emotion of trust and feeling of the robot’s commitment, perception of the robot’s reliable task fulfillment, and team fluency are associated with operators’ proficiency in performing activities recognized as part of their jobs [69] at the workstations with a cobot. In other words, this suggests that task performance (i.e., behavior) is mostly related to operators’ emotions towards and judgments of the robot, as well as the coordinated fulfillment of joint tasks with a capable and supportive agent. Hence, the result resonates with the notion that HRI fluency is a job resource [40], and, as such, it has motivating properties and facilitates operators to present expected behavior. As for the discretionary behavior promoting the effective functioning of groups [72] and the organization [63,73] as well as the creation of new products, ideas, procedures, or processes [75,76], they are mostly related to team-oriented fluency, which reflects the synergy-driven simultaneous or sequential performance of joint tasks [1] by two equally important and capable teammates, one of which is robotic and the other is human. A probable explanation for this outcome is that a supportive cobot helps the operator in the simultaneous task fulfillment, which results in operators being less fatigued and thus able and willing to engage in activities other than the mandatory ones. The other activities may include citizenship behavior, e.g., helping others or shaping the work context, and creative performance. With regard to task performance, the study results are consistent with previous investigations that showed positive associations between fluency in HRI and individual performance [31,40,79] as well as with the general notion of academics and practitioners that deploying cobots in production systems is majorly beneficial for the efficiency of employees [1,7,21] and the productivity of the system [2], provided that the interaction is designed not to neglect the importance of human factors [24,25] such as cognitions and emotions [13]. As for organizational citizenship behavior and creative performance, to the author’s best knowledge, this is the first study that verified the relationships between these facets of employee behavior and fluency in HRI. Together, this slice of research indicates that well-orchestrated and well-tuned interaction of operators and cobots is important for individuals and organizations due to its effects on mandatory task completion and goal achievement, the shaping of the organizational, social and psychological context in which tasks are performed, and bottom-up innovation through operators’ creativity.

The analysis also revealed associations between four facets of fluency in HRI (i.e., the human contribution-oriented, the human emotions-oriented, the robot-oriented, and the team-oriented fluency) and job satisfaction. As predicted, cognitive, emotional, and behavioral aspects of HRI fluency positively relate to operators’ knowledge, evaluative judgments, and good opinions about the job [53]. Although cobots have been generally promoted as an example of enabling technology [7] that helps prevent adverse health effects of work [16] and promotes workers’ well-being [1,22], this might be the first study to empirically investigate such a relationship. As such, it gives evidence of the significance of coordinated cooperation between collaborative robots and their human counterparts for the job-related satisfaction of the latter. This result suggests that cobots do not pose a realistic or symbolic threat to surveyed operators [60] who do not fear being replaced by them [52,59]. Contrarily, cobots are associated with positive cognitive evaluations, which are beneficial for employees’ functioning at work and the stability of the organizational system due to higher effort presented by satisfied workers [52] and lower turnover rates among them [57].

Within the theoretical framework of the Job Demand-Control-Support model [86,87], the job of a cobot operator is viewed as an active job [40], where high job demands meet high resources (especially high levels of control). Based on the presented research results, fluency in interacting with a cobot is the operator’s means to subjectively feel control over the situation. The feeling of control comes from the operator’s hands-on engagement and reliable input in the task fulfillment [40], his or her trust in the robot due to its reliability in contribution to the tasks [11], predictability [17], and fulfillment of expectations [45], and the synergic cooperation possible through team structure [11] and coordination [49]. Control over the situation and social support received from the collaborative robot [10,42] help counteract the adverse or challenging job demands and may lead directly to job satisfaction [86,88] for operators working in automated jobs [19] and to job performance, either directly [86] or indirectly through the enhancement of a positive affective-cognitive state of work engagement [40].

The research was also set to verify the moderating role of workload in the relationships of HRI fluency with job performance and job satisfaction. As [83] noted, the quantitative workload is an important concern for workers’ well-being and performance. Speciically, the quantitative workload was chosen because it requires cognitive, emotional, and behavioral efforts [84], and, as such, it resonates with the cognitive, emotional, and behavioral aspects of fluency in human-robot interaction [31], which is a job resource for cobot operators [40]. The moderation analysis revealed that workload indeed moderated the associations of HRI fluency with organizational citizenship behaviors, creative performance, and job satisfaction. Precisely speaking, the higher the workload, the weaker the investigated relations. The obtained results suggest that cobot deployment should be done with caution and regard for human factors, as collaborative robots can bring both benefits and physical and mental demands to human operators. An appropriate workload that does not exceed operators’ resources can challenge them positively [83,86] and facilitate fluent human-robot interactions [11,101] with its consequences due to optimal activation generated in a motivational process [83]. At the same time, an excessive workload due to the fast job pace [28] and repeatability of movements [10], the necessity to execute time-sharing activities [21], or the introduction of more complex tasks [11] colliding with operators’ preferences [31,90] may be detrimental to workers’ performance [82,85] and job attitudes [83], including job satisfaction. The adverse effects of a high quantitative workload stem from the fact that it requires operators’ physical and mental efforts to cope with it, leading to them feeling tired (in a short-term perspective) and exhausted (in a long-term perspective). These are signs of workers’ low energy and fatigue, which may prevent them from being motivated, and from being cognitively, emotionally, and behaviorally involved in fluent human-robot interactions, or capable of taking full advantage of the interaction with a collaborative robot. Nevertheless, the moderation analysis did not confirm the moderating role of workload in the relationship between HRI fluency and task performance. Regardless of the level of qualitative workload, the association is strong and positive. This result resonates with previous studies [31,40] and further supports the importance of fluent and highly coordinated cooperation between humans and robots as a job resource that allows operators to feel control at work with a cobot and fulfill their mandatory tasks, no matter how high the pace and volume of work is.

The obtained results indicate different strategies that can be employed to maximize the positive consequences of fluent human-robot interactions. First, proper training sessions for cobot operators are paramount to building cognitive and affective trust towards the robots and consequent robot acceptance, followed by high performance and satisfaction. While cognitive trust stems from the knowledge about how the robot operates, affective trust is developed more spontaneously through anthropomorphizing the device [35]. Hence, granting the operators sufficient time to learn what the robot is and how it operates helps to ease further on-the-job interactions. Furthermore, because cobots are both subjects of interaction and objects of design and deployment, it is necessary to support cooperation and communication between professionals collaborating with them and those responsible for their effective functioning. Moreover, the operators’ states during interaction with the robots should be perpetually monitored. Such strategies include periodic (e.g., weekly or monthly) surveys regarding good and problematic events at the robot stations or experience-sharing sessions for the operators to build necessary competencies by exchanging knowledge and expertise. These interventions help keep the lights on as regards employees’ perceived satisfaction and subjective workload, thus allowing for a fast reaction in case problems arise.

### Strengths and Limitations of the Study

The present study has some limitations and strengths that must be considered. First, due to the self-report design of the study, the investigated phenomena should be contemplated as subjectively perceived by the participants and may be susceptible to bias because of the respondents’ probable propensity for providing socially desirable responses. However, the study was conducted anonymously and kept confidential to mitigate this risk. Moreover, the measurement of human-robot interaction has often been done with self-report measures [102] with the notion that relying on employee’s knowledge of their behavior is valid [103] and accessible [104]. Nevertheless, future studies should aim to gather more objective data about different facets of job performance and work attitudes. Second, the study investigated the relationships of interest in a correlative design. For more robust results, future research should aim to gather data in a multi-wave and multi-source approach. Nevertheless, the study gathered data from a comparatively large group of operators [31] who interact with cobots daily, increasing the ecology and generalizability of the results. Lastly, decisions on facets of job performance, job satisfaction, and workload needed to be made to select the studied variables. However, there are other phenomena that are worth researching, such as counterproductive and adaptive employee behavior, job-related affective well-being or satisfaction, or qualitative workload. These are potentially valuable topics for subsequent studies.

## 7. Conclusions

The study, conducted among professional operators interacting with collaborative robots of several different brands, including KUKA, FANUC, Universal Robots, ABB, Kawasaki, ALVO, Yaskawa, OMRON, and Epson, identified positive relationships of cognitive, emotional, and behavioral aspects of fluency in human-robot interaction with employees’ behavior and attitudes. The empirical investigation also revealed that it is vital not to overload the cobot operators in order to take advantage of the fluent HRI and allow them to draw from it to shape their performance and satisfaction. The final take-home message is that the research on human-robot interaction should consider human factors and cognitive ergonomics to maximize the benefits and reduce the potential work- and health-related risks of cobot deployment among their human co-workers.

## Figures and Tables

**Table 1:** Descriptive statistics for study variables.

Variables	M(SD)	Min-Max	Skewness (SE = 0.17)	Kurtosis (SE = 0.34)	K-S Test
Human contribution-oriented fluency	5.56 (1.03)	3.00–7.00	−0.42	−0.30	0.09 ***
Human emotions-oriented fluency	5.55 (1.05)	1.33–7.00	−0.89	1.47	0.13 ***
Robot-oriented fluency	5.70 (0.96)	3.00–7.00	−0.58	0.14	0.13 ***
Team-oriented fluency	5.68 (1.01)	2.67–7.00	−0.66	0.19	0.11 ***
Overall human-robot interaction fluency	5.62 (0.94)	2.92–7.00	−0.49	0.16	0.07 *
Task performance	5.73 (0.94)	2.67–7.00	−0.57	0.10	0.11 ***
Organizational citizenship behavior	5.45 (1.05)	2.67–7.00	−0.23	−0.75	0.10 ***
Creative performance	5.42 (1.12)	2.33–7.00	−0.60	−0.33	0.13 ***
Job satisfaction	5.54 (0.99)	3.00–7.00	−0.44	−0.30	0.09 ***
Workload	2.74 (1.03)	1.00–5.00	−0.03	−0.85	0.08 **

Legend. M—Mean, SD—Standard Deviation, SE—Standard Error, K-S Test—Kolmogorov–Smirnov Test. Note. *n* = 200. * *p* < 0.05, ** *p* < 0.01, *** *p* < 0.001.

**Table 2:** Pearson correlation analysis among human–robot interaction fluency, task performance, organizational citizenship behavior, creative performance, and job satisfaction.

Variables	(1)	(2)	(3)	(4)	(5)	(6)	(7)	(8)
(1) Human contribution-oriented fluency	-							
(2) Human emotions-oriented fluency	0.84 ***	-						
(3) Robot-oriented fluency	0.84 ***	0.80 ***	-					
(4) Team-oriented fluency	0.81 ***	0.78 ***	0.86 ***	-				
(5) Overall human-robot interaction fluency	0.94 ***	0.92 ***	0.94 ***	0.93 ***	-			
(6) Task performance	0.74 ***	0.75 ***	0.79 ***	0.80 ***	0.83 ***	-		
(7) Organizational citizenship behavior	0.71 ***	0.69 ***	0.73 ***	0.79 ***	0.78 ***	0.78 ***	-	
(8) Creative performance	0.66 ***	0.65 ***	0.66 ***	0.73 ***	0.72 ***	0.69 ***	0.82 ***	-
(9) Job satisfaction	0.84 ***	0.81 ***	0.81 ***	0.85 ***	0.89 ***	0.82 ***	0.83 ***	0.80 ***

Note. *n* = 200. *** *p* < 0.001.

**Table 3:** Hierarchical regression analysis of human-robot interaction fluency factors as predictors of task performance, organizational citizenship behavior, creative performance, and job satisfaction.

Variables	Task Performance		Organizational Citizenship Behavior		Creative Performance		Job Satisfaction	
	B(SE)	β	B(SE)	β	B(SE)	β	B(SE)	β
Human contribution-oriented fluency	0.08 (0.08)	0.04	0.10 (0.10)	0.09	0.14 (0.11)	0.13	0.30 (0.07)	0.31 ***
Human emotions-oriented fluency	0.18 (0.07)	0.21 **	0.10 (0.08)	0.10	0.12 (0.10	0.12	0.17(0.06)	0.18 **
Robot-oriented fluency	0.24 (0.09)	0.25 **	0.13 (0.11)	0.12	−0.02 (0.13)	−0.02	0.06 (0.08)	0.06
Team-oriented fluency	0.37 (0.08)	0.40 ***	0.55 (0.10)	0.53 ***	0.61 (0.11)	0.55 ***	0.41 (0.07)	0.42 ***
*F*	111.95 ***		86.81 ***		59.72 ***		198.97 ***	
R^2^	0.70		0.64		0.55		0.80	
Adj. R^2^	0.69		0.63		0.54		0.80	

Legend. B—unstandardized regression coefficient, SE—Standard Error, β—standardized regression coefficient. Note. *n* = 200. ** *p* < 0.01, *** *p* < 0.001.

**Table 4:** Moderation analysis with overall fluency in human-robot interaction as a predictor, workload as a moderator, and task performance, organizational citizenship behavior, creative performance, and job satisfaction as dependent variables.

Variables	Task Performance			Organizational Citizenship Behavior			Creative Performance			Job Satisfaction		
	β	95% CI	t	β	95% CI	t	β	95% CI	t	β	95% CI	t
Constant	0.00	−0.08; 0.07	0.01	0.00	−0.08; 0.08	0.04	0.00	−0.09; 0.10	0.03	0.00	−0.06; 0.06	0.03
Overall Fluency (predictor)	0.81 ***	0.73; 0.89	18.96	0.72 ***	0.64; 0.81	16.17	0.66 ***	0.56; 0.76	13.05	0.85 ***	0.79; 0.92	25.91
Workload (moderator)	0.02	−0.07; 0.11	0.49	0.20 ***	0.11; 0.29	4.27	0.16 **	0.05; 0.26	2.96	0.14 ***	0.08; 0.21	4.15
*Interaction*												
Overall Fluency x Workload	−0.05	−0.14; 0.04	−1.05	−0.19 ***	−0.29; −0.09	−3.84	0.−19 **	−0.30; −0.08	−3.32	−0.13 ***	−0.19; −0.05	−3.34
*F*	141.56 ***			123.52 ***			80.76 ***			283.89 ***		
Δ*F*	1.11			14.75 ***			10.99 **			11.18 ***		
R^2^	0.68			0.65			0.55			0.81		
ΔR^2^	0.00			0.03			0.03			0.01		

Legend. CI—Confidence Interval, β—standardized regression coefficient. Note. *n* = 200. ** *p* < 0.01, *** *p* < 0.001.

## Data Availability

The data presented in this study are available on reasonable request from the corresponding author.
